# Non-replicative antibiotic resistance-free DNA vaccine encoding S and N proteins induces full protection in mice against SARS-CoV-2

**DOI:** 10.3389/fimmu.2022.1023255

**Published:** 2022-11-09

**Authors:** Pedro J. Alcolea, Jaime Larraga, Daniel Rodríguez-Martín, Ana Alonso, Francisco J. Loayza, José M. Rojas, Silvia Ruiz-García, Andrés Louloudes-Lázaro, Ana B. Carlón, Pedro J. Sánchez-Cordón, Pablo Nogales-Altozano, Natalia Redondo, Miguel Manzano, Daniel Lozano, Jesús Palomero, María Montoya, María Vallet-Regí, Verónica Martín, Noemí Sevilla, Vicente Larraga

**Affiliations:** ^1^ Laboratorio de Parasitología Molecular, Unidad de Desarrollo de Fármacos Biológicos, Inmunológicos y Químicos para la Salud Global (BICS), Departamento de Biología Celular y Molecular, Centro de Investigaciones Biológicas Margarita Salas, Consejo Superior de Investigaciones Científicas (CIBMS-CSIC), Madrid, Spain; ^2^ Grupo de Investigación en Nuevas Estrategias de Control de Patógenos Relevantes en Sanidad Animal, Centro de Investigación en Sanidad Animal (CISA-INIA-CSIC), Instituto Nacional de Investigación y Tecnología Agraria y Alimentaria, Consejo Superior de Investigaciones Científicas, Madrid, Spain; ^3^ Inmunología Viral: Terapias y Vacunas. Unidad de Desarrollo de Fármacos Biológicos, Inmunológicos y Químicos para la Salud Global (BICS), Departamento de Biomedicina Molecular, Centro de Investigaciones Biológicas Margarita Salas, Consejo Superior de Investigaciones Científicas (CIBMS-CSIC), Madrid, Spain; ^4^ Grupo de Investigación en Biomateriales Inteligentes (GIBI), Departamento de Química en Ciencias Farmacéuticas. Facultad de Farmacia. Universidad Complutense de Madrid, Instituto de Investigación Sanitaria, Hospital 12 de Octubre i+12, Centro de Investigación Biomédica en Red de Bioingeniería, Biomateriales y Nanomedicina (CIBER-BBN), Madrid, Spain; ^5^ Department of Physiology and Pharmacology. Instituto de Neurociencias de castilla y León (INCyL), Instituto de Investigación Biomédica de Salamanca (IBSAL), School of Medicine, University of Salamanca, Salamanca, Spain

**Keywords:** SARS-CoV-2, DNA vaccine, S protein, N protein, mouse model, pPAL, furin

## Abstract

SARS-CoV-2 vaccines currently in use have contributed to controlling the COVID-19 pandemic. Notwithstanding, the high mutation rate, fundamentally in the spike glycoprotein (S), is causing the emergence of new variants. Solely utilizing this antigen is a drawback that may reduce the efficacy of these vaccines. Herein we present a DNA vaccine candidate that contains the genes encoding the S and the nucleocapsid (N) proteins implemented into the non-replicative mammalian expression plasmid vector, pPAL. This plasmid lacks antibiotic resistance genes and contains an alternative selectable marker for production. The S gene sequence was modified to avoid furin cleavage (Sfs). Potent humoral and cellular immune responses were observed in C57BL/6J mice vaccinated with pPAL-Sfs + pPAL-N following a prime/boost regimen by the intramuscular route applying *in vivo* electroporation. The immunogen fully protected K18-hACE2 mice against a lethal dose (10^5^ PFU) of SARS-CoV-2. Viral replication was completely controlled in the lungs, brain, and heart of vaccinated mice. Therefore, pPAL-Sfs + pPAL-N is a promising DNA vaccine candidate for protection from COVID-19.

## Introduction

Vaccines are considered the most effective method to control the COVID-19 pandemic and have helped restore the global economy (1, 2). However, efficacy of the protective immune response induced by these vaccines against variants of concern (VOCs) is problematic (3). New VOCs present a high mutation rate in the receptor binding domain (RBD) and the N-terminal domain sequences of the SARS-CoV-2 spike glycoprotein (S). Antibody-based immunity against these VOCs is less effective compared to the original strain, Wuhan-Hu-1 (4, 5). Nonetheless, the T-cell response remains robust to these VOCs (6). T cells recognize more epitopes on the S protein than antibodies do (6). T-cell responses do not fade as quickly as antibody responses, and are, therefore, more effective at protecting from emerging variants (3, 7–9). Vaccines expressing the S protein from the Omicron variants don’t improve efficacy when compared to the original S protein. This indicates that altering the vaccine antigen to the most recent S form may not necessarily correlate with improved efficacy (3). Developing highly efficacious vaccines to prevent COVID-19 remains a global need. Vaccines that elicit an adequate cellular immune response will be necessary in the future to limit the impact of new VOCs on health systems (10). 

DNA vaccines have been used in veterinary medicine and induce specific protective immune responses against pathogens. They are easily modifiable allowing for quick testing of multiple vaccine candidates against new virus strains. One of the benefits of DNA vaccine production is that it can easily be scaled up. DNA vaccines are thermotolerant, and, consequently, cold chain maintenance is not required for long-term storage or worldwide distribution. Developing countries would benefit from this feature (11). Several DNA vaccine candidates are being tested in clinical trials against COVID-19 (12), although most contain an antibiotic resistance gene as a selectable marker for the manufacturing process (12). We have developed a new DNA vaccine candidate for the prevention of SARS-CoV-2 infection that consists of the complete S and the nucleocapsid (N) gene sequences cloned into the non-replicative antibiotic resistance gene-free pPAL plasmid (13). The S gene was modified to stabilize the protein product against furin cleavage (Sfs) and includes the RBD, which mediates virus entry into the host cell (14). The vaccine candidate, pPAL-Sfs + pPAL-N, is composed of a 1:1 mass ratio mixture of each plasmid dissolved in sterile water. We decided to include the genes in separate plasmids to avoid a single larger plasmid, which would result in reduced production yield. The pPAL plasmid vehicle includes a long CpG island (13) and does not require additional adjuvants. The N protein was chosen as it remains significantly conserved among betacoronaviruses (15). A protective role for the N protein against SARS-CoV-2 infections has been recently proposed (16). The N protein is the most abundant viral protein, highly immunogenic in coronavirus infections, and may contribute to broadening the T-cell response, improving cross-reactivity.

Therefore, we assessed the efficacy of the pPAL-Sfs + pPAL-N vaccine candidate against SARS-CoV-2 using a prime/boost administration regime by the intramuscular route followed by *in vivo* electroporation (11). Humoral and cellular immunity were evaluated in wild-type C57BL/6J mice and the level of protection against a lethal challenge of SARS-CoV-2 was assessed in the mouse line B6Cg-Tg(K18-hACE2)2Prlmn/J (17). The present study provides evidence that pPAL-Sfs + pPAL-N is a promising vaccine candidate against SARS-CoV-2 infection.

## Materials and methods

### pPAL constructs

**Figure 1:**
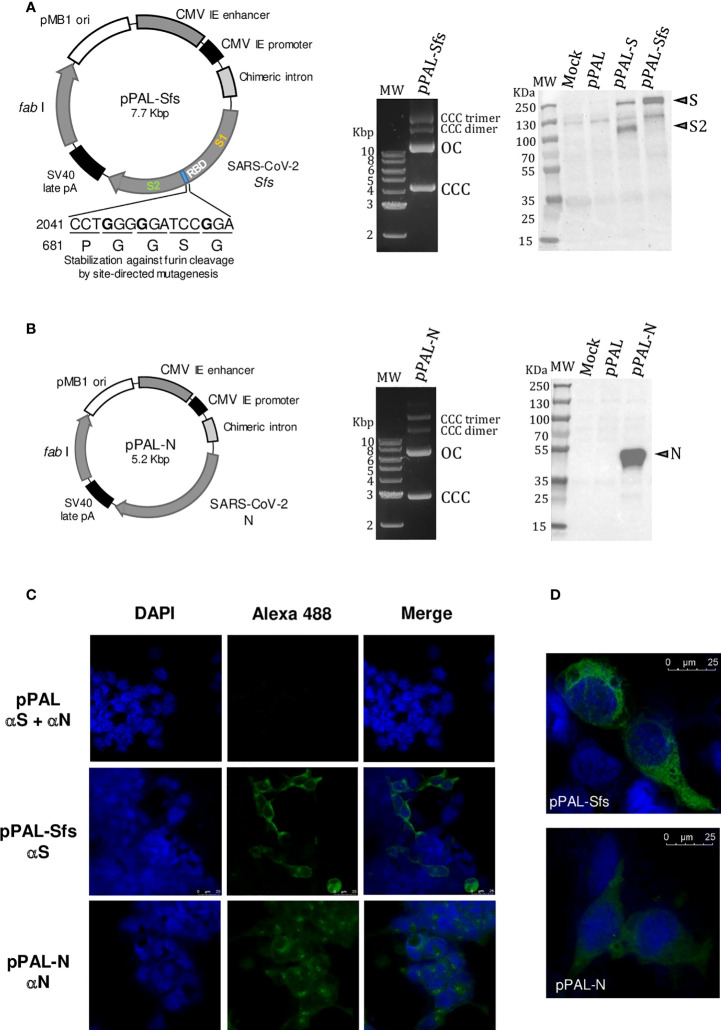
The pPAL-Sfs + pPAL-N vaccine candidate. **(A)** pPAL-Sfs map. Sfs is a codon-optimized modified version of the SARS-CoV-2 Wuhan reference sequence that contains a modification to avoid furin cleavage of the protein product. Large-scale laboratory preparation of the pPAL-Sfs plasmid containing supercoiled (CCC), open-circular (OC), CCC-dimer, and CCC-trimer conformers. Western blot of whole protein extracts from HEK293 cells transfected with pPAL, pPAL-S, and pPAL-Sfs. The primary antibody was 1:800-diluted goat anti-S polyclonal antibody (Abcam #ab272504, Cambridge, United Kingdom), which only recognizes the S2 subunit. The secondary antibody was 1:2,000-diluted HRP-conjugated goat polyclonal anti-rabbit Ig. Furin cleaves the S protein obtained from pPAL-S but not Sfs from pPAL-Sfs. **(B)** Large-scale laboratory preparation of the pPAL-N plasmid containing CCC, OC, CCC-dimer, and CCC-trimer conformers. Western blot of whole protein extracts from HEK293 cells transfected with pPAL and pPAL-N. The primary antibody was 1:500-diluted rabbit anti-N polyclonal antibody. The secondary antibody was 1:2,000-diluted HRP-conjugated goat polyclonal anti-rabbit Ig. **(C, D)** immunofluorescence of cultured HEK293 cells transfected with pPAL, pPAL-Sfs, and pPAL-N plasmids. The anti-S and anti-N antibody dilutions were 1:50. The pPAL control was incubated with both antibodies. The secondary antibody was Alexa Fluor^®^ 488-conjugated goat anti-rabbit IgG diluted to 1:200. Sfs and N expression is observed.

DNA vaccines require a vector to replicate genes and express encoding antigens. Antibiotic resistance genes are often used as selection markers, which must not be released into the environment upon final product commercialization. Considering this, the use of antibiotic resistance-free vectors is imperative. The pPAL mammalian expression plasmid vector is based on the cytomegalovirus enhancer and promoter sequences. This plasmid does not replicate in mammalian cells and does not contain selectable markers based on antibiotic resistance. The selectable marker is the *E. coli fab*I gene, which encodes for the enoyl-ACP reductase. This enzyme is inhibited by the bacteriostatic compound triclosan, which is the selection agent at an optimal concentration of 3 µM (13). The pPAL-Sfs construct (**Figure 1A**) contains a modified version of the SARS-CoV-2 S-encoding gene with NCBI acc. no. NC045512, gene ID 43740568. First, the sequence was optimized by the Monte Carlo approach according to relative codon usage frequencies. Second, the cleavage site was modified to avoid furin cleavage (PRRA → PGGS; 681-684). For this purpose, the nucleotide sequence CCTCGGCGGGCA was replaced by CCAGGCGGCAGC (2041–2052). The pPAL-N construct contains the SARS-CoV-2 N protein with NCBI acc. no. NC045512, gene ID 43740575. The KpnI-site-flanked pPAL-Sfs and pPAL-N constructs were obtained by gene synthesis in the pGH vector (ATG Biosynthetics, Merzhausen, Germany) and transferred to the *E. coli* SURE2 (Agilent, Santa Clara, CA) strain by electroporation at 1,800 V, 200 Ω, and 25 µF. Selection in LB-agar medium was performed with 3 µM triclosan (Sigma-Aldrich, St. Louis, MO, United States). pGH was excised by KpnI (NEB, Ipswich, MA) digestion, and the vaccine constructs were circularized with T4 DNA ligase (NEB). Endotoxin-free pPAL, pPAL-Sfs, and pPAL-N plasmid preparations were obtained with PureLink™ Expi Endotoxin-Free Giga Plasmid Purification Kit (Invitrogen, Waltham, MA) following the manufacturer’s instructions.

### Antigen gene expression in transfected HEK293 cells

HEK293 cells (CRL-1573™, ATCC^®^, Manassas, VA) were grown at 37 °C in a 5% CO_2_ atmosphere in complete medium (CM) containing DMEM supplemented with 10% heat-inactivated fetal bovine serum (HIFBS) (Sigma-Aldrich, Burlington, MA), 100 IU penicillin – 100 µg/mL streptomycin. Semi-confluent HEK293 cells were detached by mild pipetting and washed once with 1 mL sterile PBS and once with 0.3 mL GTporator®-M (Protean, Ceske Budejovice, Czech Republic) per 3 x 10^6^ cells. The cells were transfected by electroporation with pPAL, pPAL-S, pPAL-Sfs, or pPAL-N, and a mock transfection control that didn't contain DNA was included. Transfection of 1.2 x 10^6^ cells with 5 µg of DNA was performed at 220 V, 25 Ω, and 950 µF in 80 µL of GTporator®-M solution in a sterile 2 mm-gap cuvette (BTX, Cambridge, United Kingdom) using an ECM 630 Electro Cell Manipulator Precision Plus® (BTX, Cambridge, United Kingdom). 1.2 mL of pre-warmed medium was immediately added to the cells. 0.2 mL of the cell suspension were placed in an 8-well culture slide (Nunc^®^ Lab-Tek^®^ Chamber Slide™, Sigma-Aldrich, Burlington, MA) and 1 mL in a 24-well plate. The cell suspensions were incubated at 37 °C for 24 h in a 5% CO_2_ atmosphere.

Transfected HEK293 cells in 24-well plates were washed twice with 1 mL CM and lysed with 50 µL of a buffer containing 25 mM Tris-HCl pH 7.8, 2 mM EDTA, 2 mM DTT, 1% glycerol, and 1% Triton-X100. The protein extracts were quantified by the Bradford method. Each 20 µg protein extract was treated with 8.3 U/µL of TurboNuclease from *Serratia marcescens* (Sigma-Aldrich, Cambridge, United Kingdom) at room temperature for 10 min and prepared for SDS-PAGE in Laemmli buffer, heated at 95 °C for 5 min, and run at 30 mA for 90 min in 8-20% TGX precast SDS-PAGE gels (BioRad, Hercules, CA). Semi-dry transfer was performed on 0.45 µm nitrocellulose membranes (BioRad, Hercules, CA) at 1.3 A and 25 V for 10 min (high molecular weight transfer) in a TransBlot Turbo device (BioRad, Hercules, CA) following the manufacturer’s instructions. The membranes were blocked with 5% skimmed milk in PBS-0.1% Tween 20 (PBS-Tween) at room temperature under mild shaking for 1 h and washed thrice in PBS-Tween, 15, 5, and 5 min, respectively. The membrane was incubated at room temperature under mild shaking for 90 min with the primary antibodies prepared at the appropriate dilutions in blocking buffer. The rabbit anti-SARS-CoV-2 S polyclonal antibody #ab272504, specifically recognizing the S2 subunit (Abcam, Cambridge, United Kingdom), was used at a 1:750 dilution in blocking solution. The anti-SARS-CoV-2 N polyclonal antibody, kindly provided by Mercedes Domínguez and Inmaculada Moreno (Centro Nacional de Microbiología, Virología e Inmunología Sanitarias, Instituto de Salud Carlos III), was used at a 1:500 dilution in blocking solution. After washing three times, a 1 h incubation was carried out with the secondary HRP-conjugated goat anti-rabbit whole IgG polyclonal antibody (DAKO Agilent Technologies, Santa Clara, CA) at a 1:2,000 dilution in blocking solution. After repeating the wash steps, chemoluminescence was developed with ECL Western Blotting Reagent (Thermo Fisher Scientific, Waltham, MA) for 1 min. The images were acquired with a ChemiDoc MP Image System (BioRad, Hercules, CA). The colorimetric and the chemoluminescence images were merged using ImageLab 6.1. software (BioRad, Hercules, CA).

Transfected cells in 8-well slides were washed once with 200 µL of a hypotonic solution (11:9 water:DMEM) and twice with 1:1 acetone:methanol. Fixation and permeabilization was performed with 1:1 acetone:methanol at -20 °C for 10 min. The preparations were air-dried and the wells were carefully removed from the slides. Three 5 min washes with 0.22 µm-filtered PBS were carried out in a Coplin jar. The preparations were then air-dried and blocked with 20 µL of a 5% skimmed milk solution in 0.22 µm-filtered PBS-Tween at 37 °C in a humid chamber for 1h. After removing the excess blocking solution, 20 µL of a 1:50 dilution in blocking buffer of the anti-S2 and anti-N primary antibodies mentioned above were added to the corresponding preparation and incubated for 1 h. A single 5 min wash step was applied. Then, the cells were incubated with 20 µL of 1:200-diluted Alexa Fluor^®^ 488-conjugated goat anti-rabbit IgG secondary antibody (Jackson ImmunoResearch, West Grove, PA) at room temperature in the dark for 1 h. 10 min before the secondary antibody incubation was completed, 20 µL of 10 µg/mL DAPI in 0.22 µm-filtered PBS were added. The slides were washed four times with 0.22 µm-filtered PBS, 5 min per wash, and were then mounted with 50 µL Mowiol 4-88 and left to dry at 4 °C for 16 h in the dark. The fluorescence images were acquired with an SP8 STED 3X confocal microscope (Leica Wetzlar, Germany).

### Immunization with pPAL-Sfs + pPAL-N

87 eight-week-old wild-type C57BL/6J female mice (Charles River, Wilmington, MA) were used for immune response analysis, and 140 eight-week-old B6Cg-Tg(K18-hACE2)2Prlmn/J female mice (Jackson ImmunoResearch, West Grove, PA) were employed in protection experiments. The animals were generally lodged in groups of five, except for four groups of eight C57BL/6J mice in the dose-response experiment, always following the space requirements specified in legislation (EU Directive 2010/63 and Spain regulation RD53/2013, modified by RD1386/2018). The mandatory permits to perform the experiments were approved by the Consejo Superior de Investigaciones Científicas (CSIC) Ethics Committee and the Comunidad Autónoma de Madrid. Experimentation with infected mice was carried out in BSL3+ laboratories (CISA-INIA-CSIC). All animals received food and water *ad libitum*. Animal welfare measures were applied, considering replacement, reduction, and refinement. Environmental enrichment was implemented. Anesthesia with isofluorane (3% for induction, 1.5% for maintenance) was administered while the vaccine inoculation followed by *in vivo* electroporation were being applied. 0.2 mg/Kg ibuprofen was then added to the drinking water. The same anesthesia was administered upon sacrifice by intracardiac puncture. All other measures specified in the regulations were applied. The endpoint criterion was adopted when appropriate. In the specific case of the K18-hACE2 mice after the viral challenge, euthanasia was immediately applied when animal weight decreased 20% or more and when any incipient sign of suffering was detected. The procedures applied in challenge experiments were then subject to retrospective evaluation.

C57BL/6J and K18-hACE2 mice were immunized by the IM route with the pPAL-Sfs + pPAL-N vaccine, which is an endotoxin-free DNA mixture composed of 20 µg pPAL-Sfs and 20 µg pPAL-N in sterile CHROMASOLV™ water (Honeywell Riedel-de Haën, Charlotte, NC). Control mice received 40 µg of pPAL. Electroporation was applied using two 30G (0.3 x 13 mm) electrode needles connected to an ECM 830 Square Wave Electroporation System (BTX, Harvard Bioscience Inc., Cambridge, MA, USA), which were placed equidistant to the inoculation point in the direction of the muscle fibers, leaving a ~5 mm separation between them. The negative pole was placed in the posterior position. Six 50 ms 100 V pulses were applied in 1 s intervals. The animals were immobilized on an electrically isolated surface and were kept under isofluorane anesthesia (3% induction, 1.5% maintenance) during the procedure. 0.2 mg/Kg ibuprofen was administered in drinking water after the procedure. Wild-type C57BL/6J mice were euthanized a week after the booster dose for cellular immune response evaluation in the spleen. K18-hACE2 mice were subjected to challenge 2 weeks after the booster dose following humoral immune response evaluation.

### ELISA

100 µL of peripheral blood was collected 1 week before vaccination and 15 days post-vaccination. Blood was left to clot at 4 °C for 5 h. Serum samples were obtained by centrifugation at 8,000 x*g* for 10 min and stored at -20 °C until use. Serial dilutions (1/3) of sera were prepared with 1% BSA in PBS-0.05% Tween 20. 96-well flat-bottom microplates were coated with 50 μL of 50 μg/mL recombinant S1+S2 ECD-His protein (Sino Biological, Beijing, China) and RBD (RayBiotech Life, Peachtree Corners, GA) in 3.36 mM carbonate–10 mM bicarbonate buffer at 4° C for 16 h. After three washes with 1% BSA in PBS-0.05% Tween 20, blocking was performed in PBS-0.05% Tween 20 containing 3% BSA for 1 h at room temperature. Next, the washes were repeated, and 100 μL of diluted serum samples were added, incubating for 1 h. After repeating the wash step, incubations with 1:8,000-diluted HRP-conjugated protein A (Invitrogen, Waltham, MA), 1:20,000 goat anti-mouse IgG1, or 1:20,000 goat anti-mouse IgG2c (Bethyl Laboratories, Montgomery, TX) were performed for 1 h, followed by three final washes. Color development was executed with TMB Substrate Kit (Thermo Scientific, Waltham, MA) for 10 min in 100 µL. The reactions were stopped by adding 100 μL of 2 N H_2_SO_4_. A_450_ was registered with Microplate Reader 680 (BioRad, Hercules, CA) and Microplate Manager 5.2.1 software (BioRad, Hercules, CA).

### SARS-CoV-2 neutralization assays

The SARS-CoV-2 MAD6 viral strain and B.1.617.2 (Delta) variant were kindly provided by Dr. Luis Enjuanes and Dr. Juan Francisco García-Arriaza (CNB-CSIC, Madrid, Spain), respectively. The MAD6 SARS-CoV-2 genome sequence is identical to the Wuhan-Hu-1 isolate (GenBank MN908947). Serial dilutions of heat-inactivated mouse sera (starting at 1:10) were incubated with 100 PFU of SARS-CoV-2 MAD6 or B.1.617.2 (Delta) for 1 h at 37 °C in 96-well flat-bottom plates. 2×10^4^ Vero E6 cells per well were then seeded on the sera/virus mixture and incubated for 3 days at 37 °C, 5% CO_2_. Culture media was then removed, and cells were fixed with 2% paraformaldehyde prior to staining with 2% crystal violet. Neutralizing antibody (NAb) titers were calculated as the serum dilution at which less than 50% cytopathic effects were observed in replicate wells.

### ELISpot and intracellular cytokine staining

Splenocyte suspensions were obtained in 15 mL of PBS containing 0.3 mM EDTA and 2% HIFBS (wash solution) using 0.45 µm BD Falcon Cell Strainers (BD Biosciences, San Jose, CA). The cells were harvested by centrifugation at 500 x*g* for 7 min and erythrocytes were lysed at room temperature for 2 min with 5 mL of 1X RBC Lysis Buffer (pluriSelect Life Science, Leipzig, Germany). 25 mL of wash solution were immediately added, and the cells were centrifuged at 500 x*g* for 7 min. After an additional wash, the cells were resuspended in 5 mL of proliferation medium containing RPMI supplemented with 10% HIFBS, 100 UI penicillin – 100 µg/mL streptomycin, and 4.5 µM β-mercaptoethanol. The cell suspensions were filtered through 0.45 µm BD Falcon Cell Strainers (BD Biosciences, San Jose, CA) and an aliquot was 1:1 diluted with 0.4% Trypan Blue solution (Sigma-Aldrich, St. Louis, MO) for live cell counting in TC10 Cell Counting Slides using a TC10 Automated Cell Counter (BioRad, Hercules, CA).

ELISpot assays were performed with the Murine IFN-γ ELISpot Kit (Abcam, Cambridge, United Kingdom) following the manufacturer’s instructions. In the stimulation step, 2 x 10^5^ splenocytes were seeded in 200 µL of proliferation medium containing the appropriate stimulus. Stimulation was performed in triplicate at 37 °C for 20 h in a 5% CO_2_ atmosphere. The stimuli were: i) 1 µg/mL concanavalin A (Sigma-Aldrich, St. Louis, MO); ii) an equimolar peptide mixture at a final concentration of 25 µg/mL representing the whole S protein sequence prepared from the PepTivator^®^ SARS-CoV-2 Prot_S1 and PepTivator^®^ SARS-CoV-2 Prot_S (Miltenyi Biotec, Bergisch Gladbach, Germany) mixtures; and iii) an equimolar peptide mixture representing the whole N protein sequence at a final concentration of 25 µg/mL prepared from the PepTivator^®^ SARS-CoV-2 Prot_N (Miltenyi Biotec, Bergisch Gladbach, Germany) mixture.

Splenocytes were plated in 96-well flat-bottom tissue culture plates (1x10^6^ per well) and stimulated with S peptide pool (25 μg/mL) (Peptivator^®^ SARS-CoV-2 Prot_S Miltenyi Biotec, Bergisch Gladbach, Germany), or with phorbol 12-myristate 13-acetate (50 ng/mL) (Sigma-Aldrich, St. Louis, MO) and ionomycin (1 μg/mL) (Sigma-Aldrich, St. Louis, MO) as the positive control, or left unstimulated as the negative control. Brefeldin-A (5 μg/mL), monensin (2 μM), and anti-CD107a antibody (1 μg/mL) (PE anti-mouse CD107a; clone 1D4B) (Biolegend, San Diego, CA) were also added to all wells at this stage. Splenocytes were incubated for a further 4 h in a humidified incubator at 37 °C, 5% CO_2_. Cells were then washed with PBS and stained with viability marker LIVE/DEAD™ Fixable Near-IR Dead Cell Stain Kit (Thermofisher, Waltham, MA) as described in the manufacturer’s protocol for 20 min on ice. Cells were then washed in staining buffer (PBS + 2% HIFBS + 0.02% sodium azide) and stained for surface cell markers (Alexa Fluor^®^ 488 anti-mouse CD8a, clone 53-6.7; Brilliant Violet 510™ anti-mouse CD4, clone GK1.5; and PerCP/Cyanine5.5 anti-mouse CD3, clone 17A2; all from Biolegend, San Diego, CA) for 20 min on ice. Cells were washed twice in staining buffer, and fixed and permeabilized using the Cytofix/Cytoperm Fixation/Permeabilization kit (BD Biosciences, San Jose, CA) according to the manufacturer’s instructions for 20 min on ice, followed by staining for intracellular cytokines for 30 min on ice (Brilliant Violet 421™ anti-mouse TNF-α, clone MP6-XT22; Alexa Fluor^®^ 647 anti-mouse IFN-γ, clone XMG1.2; and Brilliant Violet 785™ anti-mouse IL-2; all from Biolegend, San Diego, CA). After washing as indicated in the Cytofix/Cytoperm Fixation/Permeabilization kit, cells were resuspended in staining buffer and acquired with a FACSCelestaSORP flow cytometer (BD Biosciences, San Jose, CA). Data analysis was performed with FlowJo Software (BD Biosciences, San Jose, CA).

### SARS-CoV-2 maintenance and infectious challenge

The Calu3 cell line was grown in DMEM medium supplemented with 20% HIFBS and 10 mM HEPES. This cell line was kindly provided by Luis Enjuanes (CNB-CSIC, Madrid, Spain) and was used for viral propagation at a multiplicity of infection (MOI) of 0.001 PFU per cell. The supernatant was harvested 72 h after infection (hpi), subjected to three freeze/thaw cycles, and clarified by centrifugation at 1,970 x*g* for 10 min. The virus was titrated and stored at -80 °C until use. Titration was performed using the Vero E6 cell line (CRL-1586, ATCC^®^, Manassas, VA), which was maintained in DMEM medium supplemented with 5% HIFBS. Viral adsorption was allowed for 1 h in 70-80% confluent Vero E6 cell culture monolayers. 2 mL of 0.5% semisolid agar in DMEM medium supplemented with 2% HIFBS were then added. The PFU count was performed after 6 days.

Mice were challenged with 10^5^ PFU of MAD6 or Delta SARS-CoV-2 by the intranasal route 15 days after the booster dose. Thereafter, body weight and clinical profiles were followed daily. Body weight and clinical score follow-up included 10 K18-hACE2 mice per group (pPAL control and pPAL-Sfs + pPAL-N) for each experiment. The clinical score was calculated as explained in **Supplementary Table 1**. 20 mice per group in each experiment were employed for viral burden evaluation in subgroups of five on days 2, 4, 7, and 14 post-challenge.

### Clinical score evaluation

Mice were observed and weighed daily post-challenge, and clinical signs were scored according to **Table S1**. The sum score in clinical signs (based on body weight, appearance, motility, and respiration) was used to evaluate disease severity. A humane endpoint was implemented when this score reached >50 to reduce animal suffering.

### Viral load evaluation in target organs

Samples of the target organs (lung, heart, and brain) obtained on days 2, 4, 7, and 14 post-challenge were lysed applying three freeze-thaw cycles and 20 sonication cycles/min at 5 W for 2 min. The lysates were centrifuged at 220 x*g* for 5 min to obtain clarified viral stocks. Total RNA extraction was performed with Trizol Reagent (Invitrogen, Waltham, MA) following the manufacturer’s instructions. Two-step qRT-PCR was performed using SuperScript III reverse transcriptase (Invitrogen, Waltham, MA) and EHF DNA polymerase (Roche, Basel, Switzerland) as described by Toussaint et al. (18) using the N1-F (GACCCCAAAATCAGCGAAAT) and N1-R (TCTGGTTACTGCCAGTTGAATCTG) primers, and the N1-P (FAM-ACCCCGCATTACGTTTGGTGGACC-BHQ1) probe. The primers and probe for the β-actin reference gene were ACT_F_1005-1029 (CAGCACAATGAAGATCAAGATCATC), ACT_R_1135-1114 (CGGACTCATCGTACTCCTGCTT), and ACT_P_1081-1105 (JOE-TCGCTGTCCACCTTCCAGCAGATGT-BHQ1). The same clarified viral stocks from target organs were used to titrate viral replication by plaque assays in VERO cells measured as PFU/g tissue.

### Statistical analysis

Two-way ANOVA with Fisher’s LD *post-hoc* test was applied to intracellular cytokine staining experiments only. In all other experiments, statistical inference was performed with the Student’s t-test applying p-value adjustment by the Holm-Sidak method.

## Results

### HEK293 cells transfected with the pPAL-Sfs + pPAL-N vaccine candidate expresses the SARS-CoV-2 Sfs and N genes

S and N protein gene sequences were retrieved from the SARS-CoV-2 Wuhan-1 isolate genome sequence (GenBank Acc. No. MN908947). Sfs is a modified version of the S gene generated to avoid furin cleavage (2041 CCTCGGCGGGCA → CCAGGCGGCAGC; 681 PRRA → PGGS). Endogenous furin from HEK293 cells only cleaves the S glycoprotein and not Sfs as the S2 fragment is only observed in pPAL-S transfectants (**Figure 1A**). The Sfs and the N clones were separately cloned into the pPAL mammalian expression plasmid vector under the control of the CMV enhancer/promoter (13), obtaining the pPAL-Sfs + pPAL-N vaccine candidate (**Figure 1**). HEK293 cells transfected with each DNA construct express the respective antigen genes according to Western blot (**Figures 1A, B**). Indirect immunofluorescence experiments using anti-S2 and anti-N polyclonal antibodies confirmed Sfs and N expression (**Figures 1C, D**).

### pPAL-Sfs + pPAL-N vaccination induces SARS-CoV-2-specific humoral and cellular immune responses in mice

**Figure 2:**
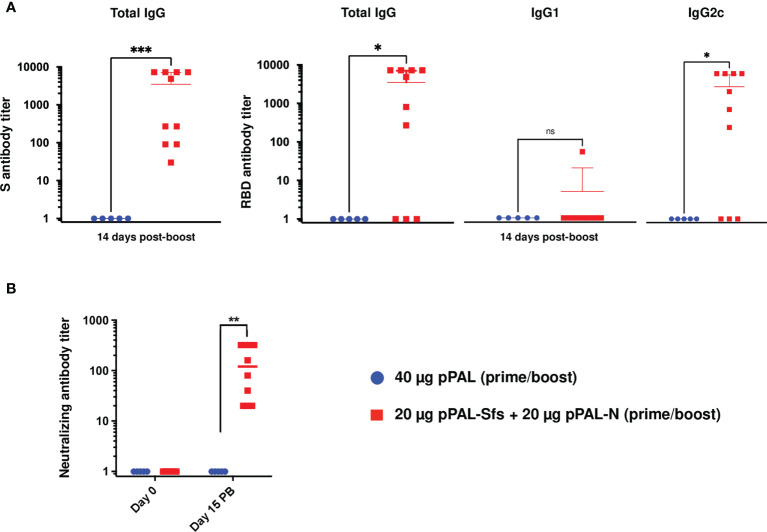
The pPAL-Sfs + pPAL-N vaccine candidates activate SARS-CoV-2 S- and RBD-specific humoral immunity in C57BL/6J mice. The serum samples were collected 15 days after the booster dose. **(A)** ELISA determinations of individual and average titers of total circulating anti-S and anti-RBD IgG determined by ELISA using HRP-conjugated protein **(A)** ELISA determinations of individual and average titers of circulating anti-RBD IgG, IgG1, and IgG2c using HRP-conjugated goat anti-mouse IgG1, anti-IgG2, and anti-IgG2c. The average anti-S and anti-RBD IgG titers are ≥2,000. The IgG2c/IgG1 ratio is ~750 in vaccinated animals with respect to the pPAL control group. **(B)** The neutralizing antibody titer average is >100. Statistical inference was performed using the Student’s t-test applying p-value adjustment by the Holm-Sidak method (α = 0.05; *p<0.05; **p<0.01; ***p<0.001; ns, non-significant).

C57BL/6J (B6) mice were immunized with pPAL-Sfs + pPAL-N (20 μg each) by the intramuscular route by applying an *in vivo* electroporation procedure following a 15-day-interval prime-boost homologous regimen. The average titers of circulating anti-S IgG, anti-RBD IgG, and anti-RBD IgG2c were >2,000. The IgG2c/IgG1 ratio was ~750 in pPAL-Sfs + pPAL-N vaccinated mice (**Figure 2A**). All vaccinated animals were positive for neutralizing antibodies (NAb). The average NAb titers were ~100 after the booster dose (**Figure 2B**). Hence, the pPAL-Sfs + pPAL-N vaccine candidate elicits a SARS-CoV-2-specific neutralizing humoral immune response in mice.

**Figure 3:**
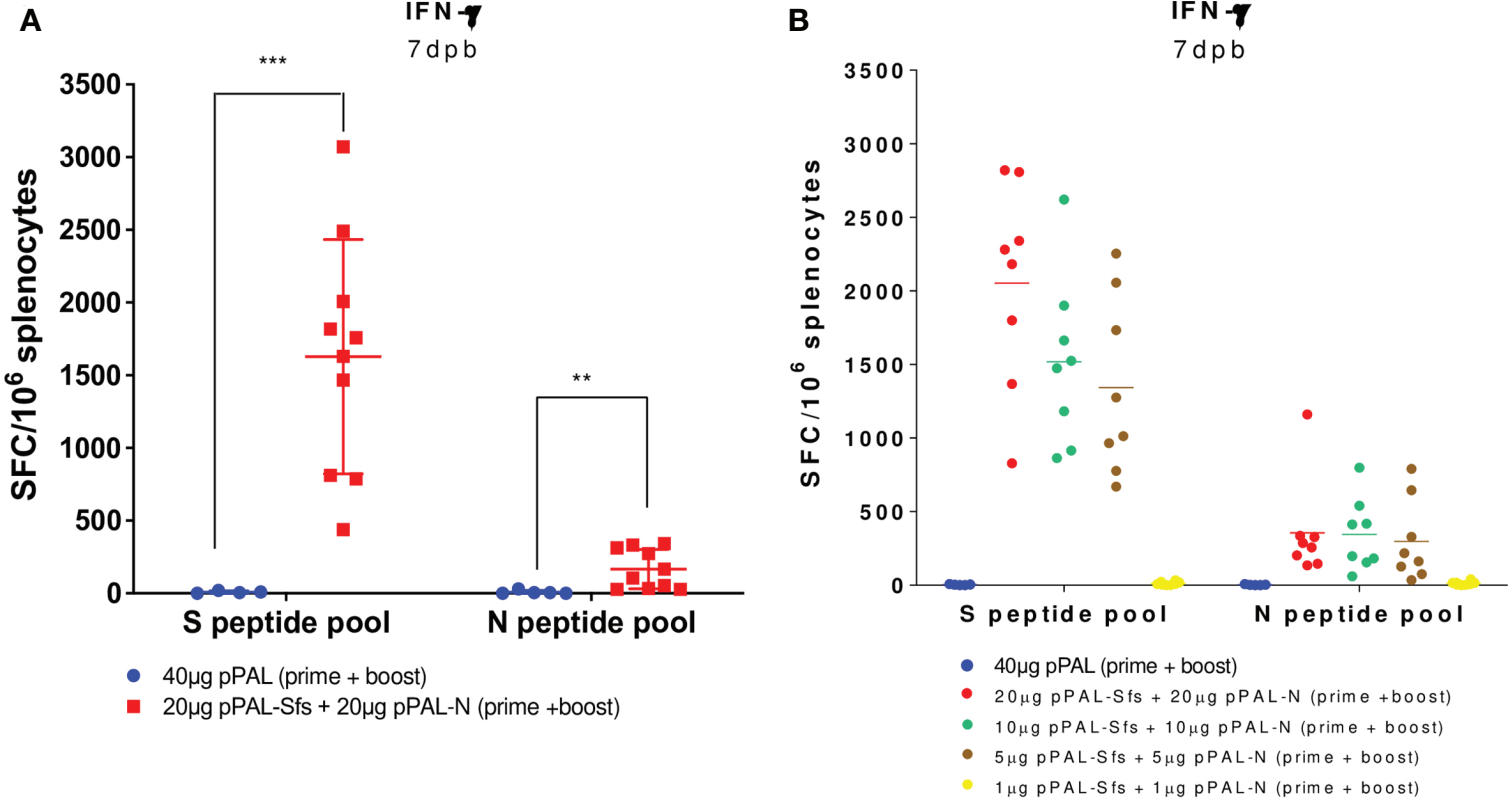
S- and N-specific activation of IFN-γ-producing splenocytes induced by the pPAL-Sfs + pPAL-N vaccine candidate in C57BL/6J mice is dose-dependent. Splenocytes were obtained 7 days post-boost after prime/boost inoculation of pPAL and pPAL-Sfs + pPAL-N. Splenocytes (2x10^5^ cells/well) were stimulated at 37 °C in IFN-γ ELISpot plates for 16 h with 11-mer 8 amino acid-overlap peptide pools (25 µg/well) representing the whole S and N amino acid sequences in equimolar amounts. Negative stimulation controls were subtracted from the SFC per million cell values. Statistical inference was performed using the Student’s t-test applying p-value adjustment by the Holm-Sidak method (α = 0.05; **p<0.01; ***p<0.001). **(A)** Robust S- and N-specific activation of IFN-γ-producing splenocytes is observed with a prime/boost immunization regime with pPAL-Sfs + pPAL-N. **(B)** S-specific activation of IFN-γ-producing splenocytes with pPAL-Sfs + pPAL-N is dose dependent.

**Figure 4:**
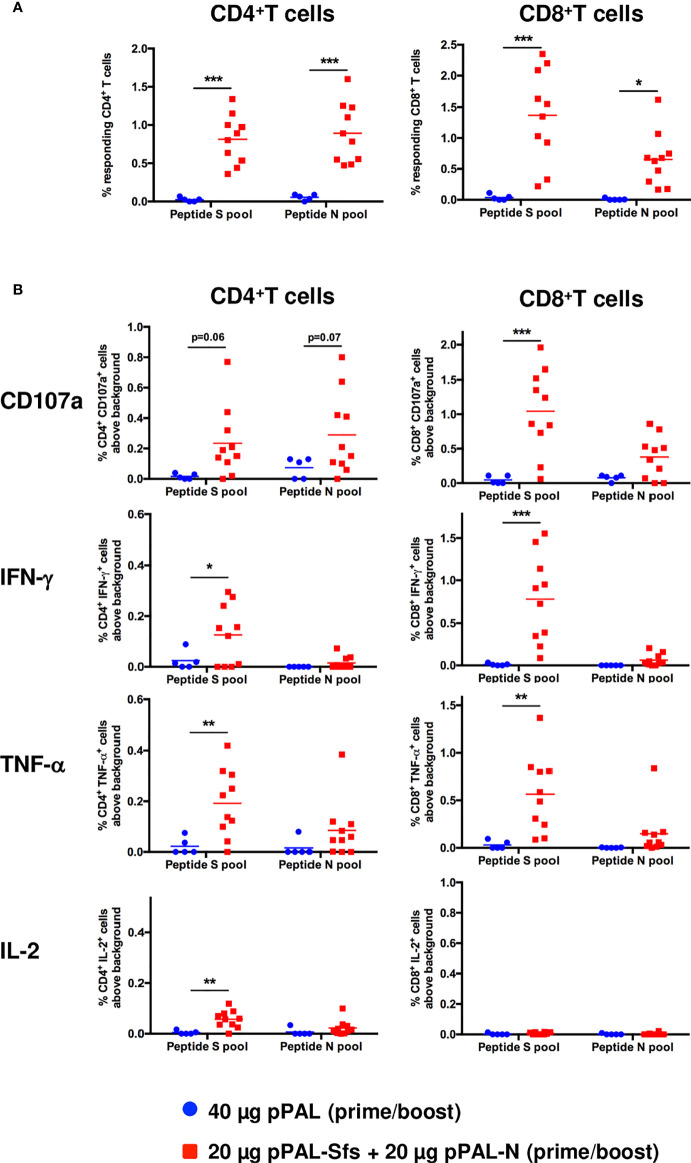
Specific CD3^+^CD4^+^ and CD3^+^CD8^+^ T-cell response induced by pPAL-Sfs + pPAL-N in C57BL/6J mice. Splenocytes were obtained from mice after prime/boost inoculation with pPAL and pPAL-Sfs + pPAL-N. Splenocytes were stimulated for 4 h with 11-mer 8 amino acid-overlap peptide pools representing the whole S and N amino acid sequences in equimolar amounts. **(A)** Percentage of CD3^+^CD4^+^ and CD3^+^CD8^+^ T cells producing CD107a, IFN-γ, TNF-α, and IL-2 in response to stimulation with peptide S or N pools. **(B)** Percentage of CD4^+^ and CD8^+^ T cells responding to S and N peptide pools above background, calculated as the sum of T cells positive for CD107a and/or IFN-γ and/or TNF-α and/or IL-2. Statistical inference using Two-way ANOVA with Fisher’s LD *post-hoc* test (*p<0.05; **p<0.01; ***p<0.001).

**Figure 5:**
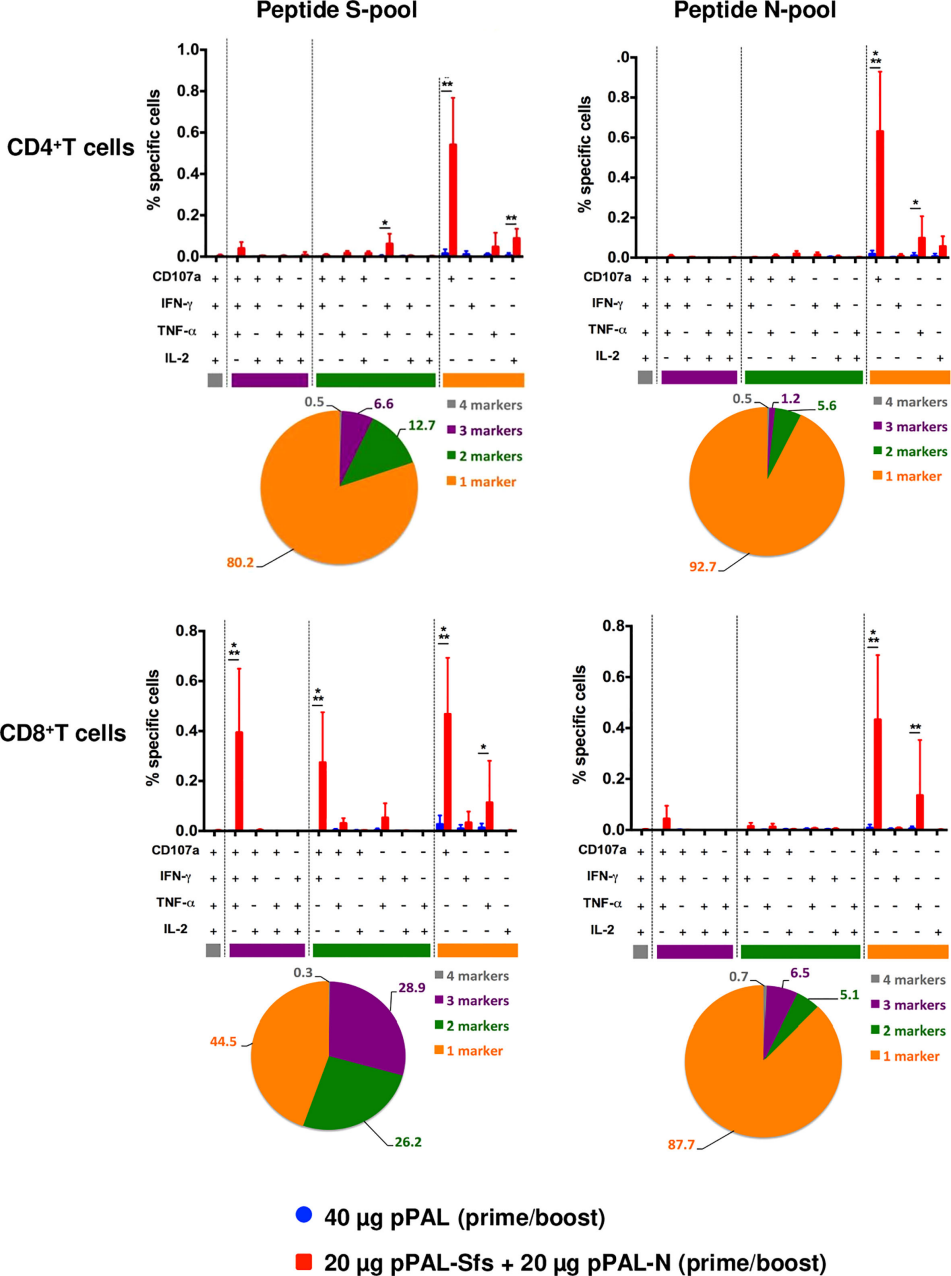
Polyfunctionality analysis of specific CD3^+^CD4^+^ and CD3^+^CD8^+^ T cell populations stimulated by pPAL-Sfs + pPAL-N immunization in C57BL/6J mice. Analyses of concomitant expression of CD107a, IFN-γ, TNF-α, and IL-2 in CD3^+^CD4^+^ T cells and CD3^+^CD8^+^ T cells in response to N peptide pool or S peptide pool stimulation. Pie charts split the percentage of T cells expressing either 1, 2, 3, or 4 markers upon stimulation. Statistical inference using Two-way ANOVA with Fisher’s LD *post-hoc* test (*p<0.05; **p<0.01; ***p<0.001).

According to an ELISpot assay, all vaccinated mice produced >500 IFN-γ-secreting spot-forming colonies (SFC) per million splenocytes on day 7 post-boost, specifically against an 11-mer peptide pool representing the whole SARS-CoV-2 S protein sequence with an overlap of 7 amino acids. The average was ~1,600, and the maximum was ~3,100 (**Figure 3A**). The mean of IFN-γ-secreting SFC per million splenocytes specific to a peptide pool representing the whole SARS-CoV-2 N protein sequence with the same characteristics is much lower (~200) (**Figure 3A**). A second independent ELISpot assay confirmed high levels of IFN-γ-secreting splenocyte clones and showed that pPAL-Sfs + pPAL-N response is dose-dependent (>2,000, ~1,500, ~1,300, and 0 in mice immunized with 20, 10, 5, and 1 μg of each plasmid, respectively) (**Figure 3B**). The T-cell response was assessed by intracellular cytokine staining (ICS). The CD107a, IFN-γ, IL-2, and TNF-α markers were monitored in CD4^+^ and CD8^+^ T cell populations in splenocyte preparations stimulated with the S and N peptide pools. All vaccinated mice produced CD4^+^ and CD8^+^ T cells stimulated specifically against the S and N peptide pools (**Figure 4A**). CD4^+^ and CD8^+^ T cell populations activated against the S peptide pool for the CD107a, IFN-γ, IL-2, and TNF-α markers were registered, except for IL-2 in CD8^+^ T cells (**Figure 4B**). Activation against the N peptide pool was lower compared to the S peptide pool for all markers, except for CD107a in CD4^+^ T cells. Particularly, no activation against the N peptides was observed for IFN-γ and IL-2 (**Figure 4B**). CD8^+^ T cells showed higher polyfunctionality than CD4^+^ T cells. Patients positive for CD8^+^ T cells are less sensitive to reinfection (19), highlighting the need for vaccines eliciting a potent cellular immune response. Approximately 30% of CD8^+^ T cells were positive for three markers (**Figure 5**). S-specific CD8^+^ T cells expressing CD107a, IFN-γ, and TNF-α were the most abundant. In summary, vaccination with pPAL-Sfs + pPAL-N triggers robust T-cell activation including Th1, cytotoxic CD4^+^, and polyfunctional cytotoxic CD8^+^ T cell populations in vaccinated animals. The presence of cytotoxic CD4^+^ T cells in vaccinated mice is consistent with previous studies (20).

### pPAL-Sfs + pPAL-N vaccination induces complete protection in K18-hACE2 mice

**Figure 6:**
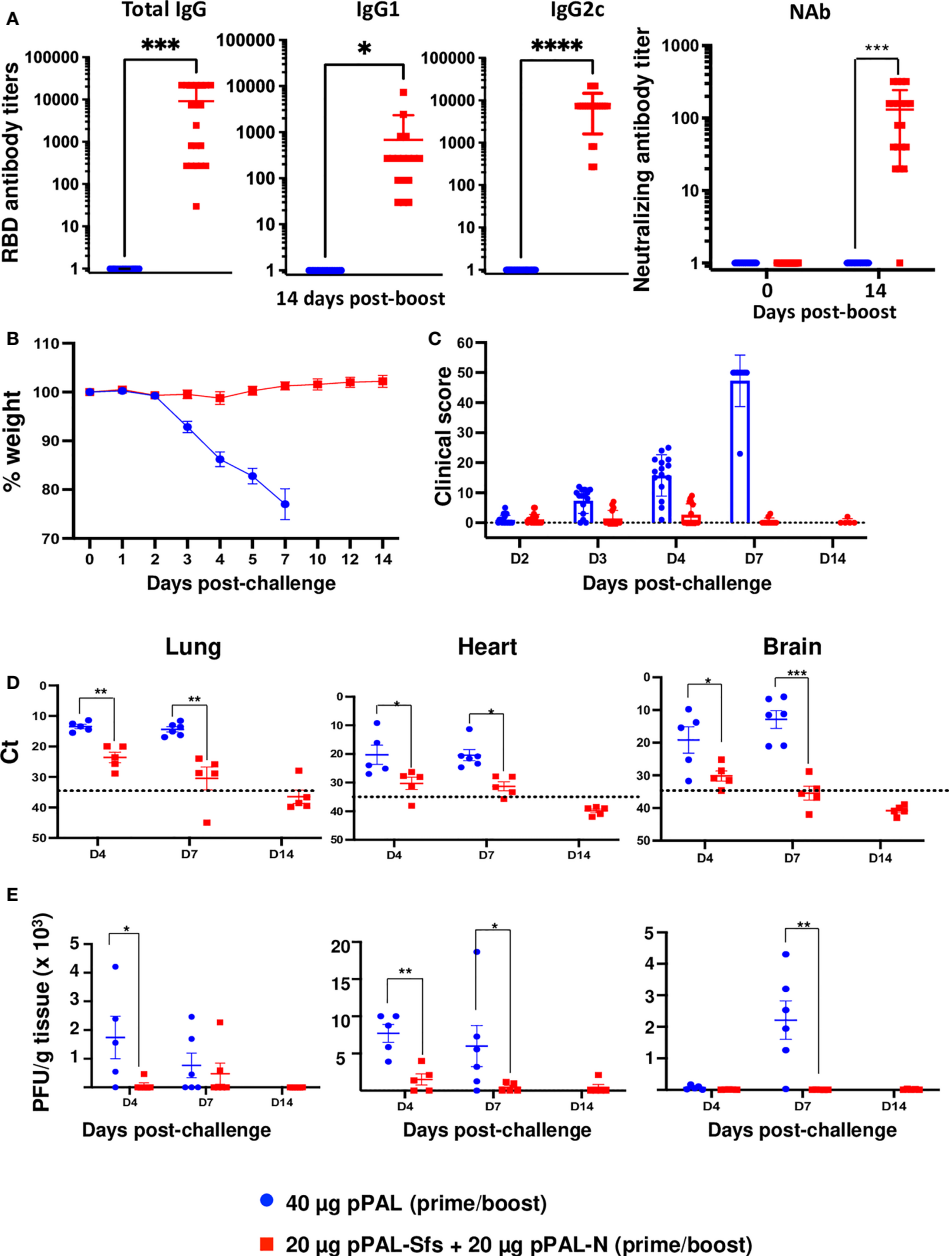
The pPAL-Sfs + pPAL-N vaccine confers full protection against challenge with 10^5^ PFU of the MAD6 SARS-CoV-2 isolate in the K18-hACE2 murine model. **(A)** Titration of circulating anti-RBD IgG, IgG1, IgG2c, and neutralizing antibodies 15 days after prime/boost immunization with pPAL-Sfs + pPAL-N. **(B)** Body weight evolution after 10^5^ PFU SARS-CoV-2 challenge. Vaccinated animals maintained their weight throughout the experiment. Weight decreased in the control group until the endpoint criteria had to be applied (20% of weight loss). **(C)** Clinical sign follow-up after challenge. **(D)** Reduction of the viral load in target organs. ΔCt accounting for SARS-CoV-2 mRNA levels. β-actin was the reference gene. **(E)** Reduction of viral replication (PFU/g tissue) in VERO cells. Statistical inference was performed using the Student’s t-test applying p-value adjustment by the Holm-Sidak method (α = 0.05; *p<0.05; **p<0.01; ***p<0.001; ****p<0.0001).

The pPAL-Sfs + pPAL-N vaccine candidate induces a specific immune response against SARS-CoV-2. Therefore, a protection efficacy experiment was performed in B6Cg-Tg(K18-hACE2)2Prlmn/J mice following the same vaccination protocol as for WT B6 mice (see below). 15 days after the booster dose, all vaccinated animals were positive for total anti-RBD IgG, IgG1, and IgG2c, with high average titers (~10,000, ~600, and ~7,000, respectively). The IgG2c/IgG1 titer ratio was ~12 (**Figure 6A**). SARS-CoV-2 antibody neutralization titers were also high except in one mouse (**Figure 6A**). Therefore, the animals were challenged with a lethal dose (10^5^ PFU) of the SARS-CoV-2 MAD6 isolate intranasally. Clinical signs and body weight were monitored after the challenge to evaluate protection. Vaccinated mice did not show any weight loss compared with control mice, which started to lose weight by day 3 post-challenge (**Figure 6B**). Furthermore, clinical signs were significantly reduced in vaccinated mice, whereas the clinical score of non-vaccinated controls increased on day 3 post-challenge (**Figure 6C**). All control mice were sacrificed by day 7 post-challenge (**Figure 6B**). Hence, complete protection was achieved in mice vaccinated with two doses of the pPAL-Sfs + pPAL-N plasmid mixture (20 μg each) administered by the intramuscular route applying *in vivo* electroporation.

Subgroups of five mice were sacrificed on day 4, day 7, and day 14 post-challenge to quantify viral replication levels. Lungs, heart, and brain were collected from each mouse to evaluate viral load by qRT-PCR and to titrate viral replication by plaque assay. Vaccinated mice showed a significant reduction in SARS-CoV-2 RNA levels in all evaluated organs compared with control mice (**Figure 6D**). The Ct values in target organs notably decreased in most vaccinated mice by day 4 post-challenge, and no viral RNA was detected on day 14 post-challenge. Quantification of replicative infectious virus in the different tissues indicated a significant reduction in vaccinated mice, with undetectable levels in most cases (**Figure 6E**). Infectious virus was only detected in the lungs of 2 out of 5 mice on day 7 post-challenge and in the hearts of 3 out of 5 mice on day 4 post-challenge. However, viral load was significantly lower compared to control animals (2.2 x 10^3^ in vaccinated mice versus 9 x 10^3^ PFU/g). The levels of infectious virus in the brain were considerably higher in control mice. The data indicates that there was initial viral replication in the lungs of control animals, followed by dissemination to the heart and brain, whereas vaccination completely controlled viral replication.

**Figure 7:**
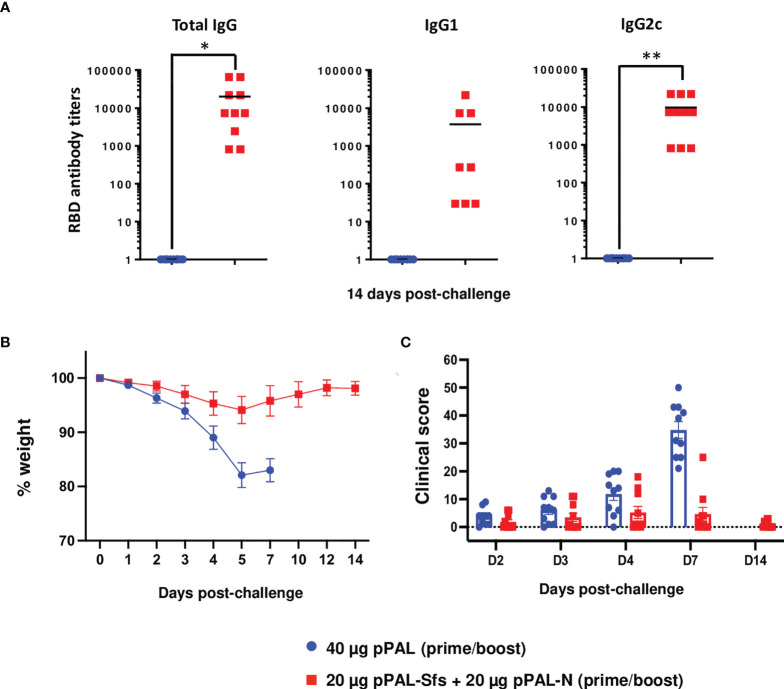
Full protection of K18-hACE2 mice after three months of vaccination with pPAL-Sfs+ pPAL-N against challenge with 10^5^ PFU of the MAD6 SARS-CoV-2 isolate in the K18-hACE2 murine model. **(A)** Titration of circulating anti-RBD IgG, IgG1, and IgG2c three months after the pPAL-Sfs + pPAL-N booster dose administration. **(B)** Body weight evolution after 10^5^ PFU SARS-CoV-2 challenge. Vaccinated animals maintained their weight throughout the experiment, whereas weight decreased in the control group. **(C)** Clinical sign follow-up after challenge with 10^5^ PFU SARS-CoV-2. Statistical inference was performed using the Student’s t-test applying p-value adjustment by the Holm-Sidak method (α = 0.05; *p<0.05; **p<0.01).

An important issue regarding new SARS-CoV-2 vaccines is long-term memory (21). To evaluate long-term protection, K18-hACE2 mice were vaccinated with two doses of 20 μg of pPAL-Sfs + 20 μg of pPAL-N with a 15-day interval between prime and boost, and lethally challenged with SARS-CoV-2 3 months after the booster dose. Antibody titers were still significantly higher in vaccinated mice compared with control mice (**Figure 7A**) after 3 months, with a value of IgG2c higher than IgG1, indicating a Th1 immune response. Protection was determined by daily registration of weight and clinical signs. Control mice lost weight continuously during the 7-day period post-challenge, and all had to be sacrificed by day 7. On the contrary, pPAL-Sfs + pPAL-N vaccinated mice did not lose weight post-challenge (**Figure 7B**). Furthermore, the clinical score was significantly higher in the control group than in the vaccinated group (**Figure 7C**). These data indicate that the pPAL-Sfs + pPAL-N vaccine induces long-term immunity in mice and, accordingly, confers protection.

### pPAL-Sfs + pPAL-N vaccination is effective against the dominant B.1.617.2 (Delta) variant

**Figure 8:**
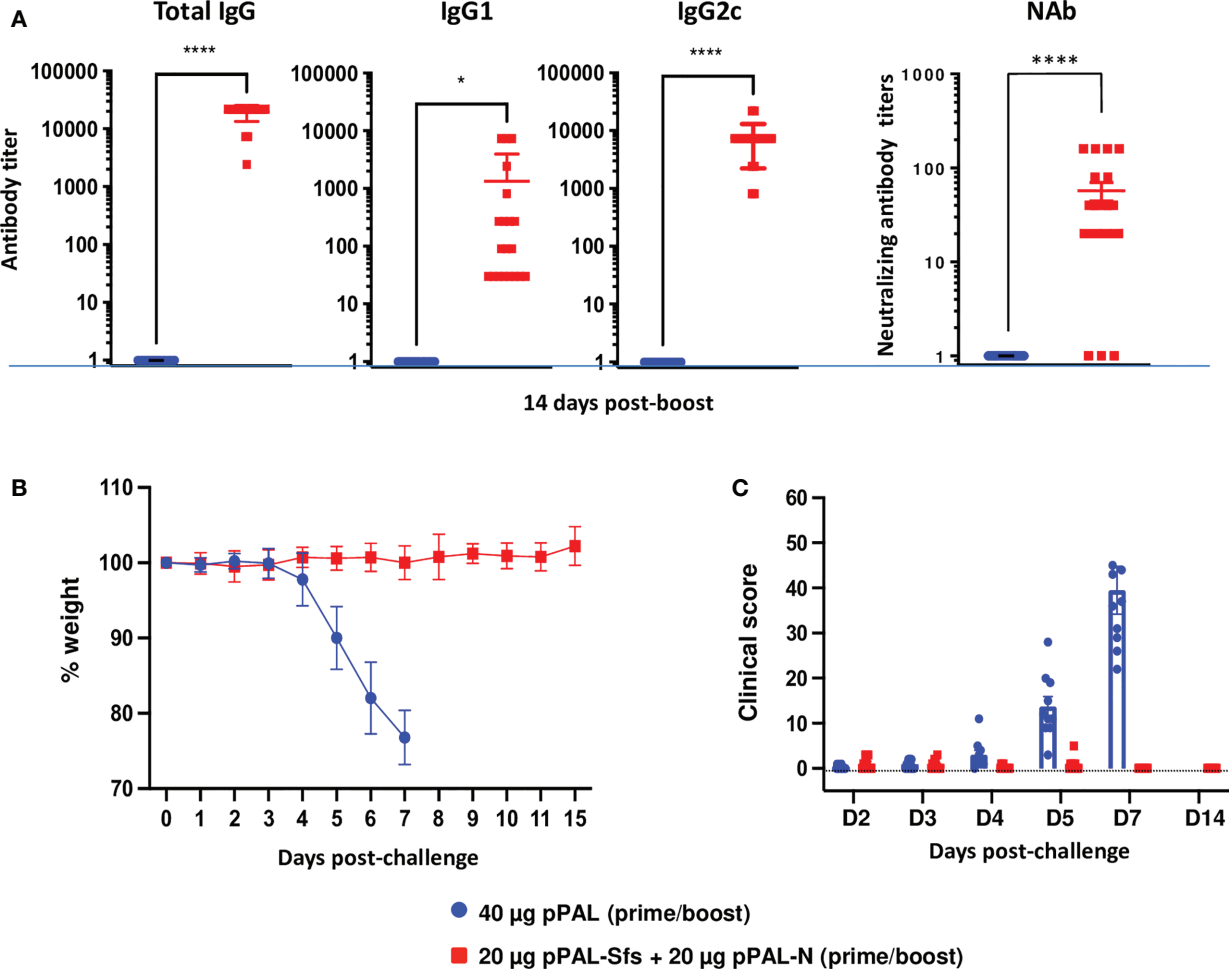
The pPAL-Sfs + pPAL-N vaccine confers full protection against challenge with 10^5^ PFU of the SARS-CoV-2 Delta variant in the K18-hACE2 murine model. **(A)** Titration of circulating anti-RBD IgG, IgG2c, and neutralizing antibodies 15 days after prime/boost immunization with pPAL-Sfs + pPAL-N. **(B)** Body weight evolution after 10^5^ PFU SARS-CoV-2 challenge. Vaccinated animals maintained their weight throughout the experiment, whereas weight decreased in the control group. **(C)** Clinical sign follow-up after challenge with 10^5^ PFU SARS-CoV-2. Statistical inference was performed using the Student’s t-test applying p-value adjustment by the Holm-Sidak method (α = 0.05; *p<0.05; ****p<0.0001).

Efficacy of the same prime-boost pPAL-Sfs + pPAL-N vaccination protocol was evaluated in K18-hACE2 mice against 10^5^ PFU of the B.1.617.2 (Delta) variant. The circulating anti-RBD IgG, IgG1, and IgG2c titers were high in vaccinated animals (>10,000, ~1,000, and 8,000, respectively). The IgG2c/IgG1 ratio was ~8, suggesting a Th1 immune response (**Figure 8A**). Weight and clinical signs were monitored daily after challenge with the SARS-CoV-2 Delta variant. All vaccinated mice maintained their initial weight, whereas the control group continuously lost weight post-challenge (**Figure 8B**). Vaccinated mice did not show significant clinical signs compared with the control group (**Figure 8C**). Therefore, the pPAL-Sfs + pPAL-N vaccine protected mice against a lethal challenge with the Delta variant.

## Discussion

The COVID-19 pandemic continues to be a primary concern worldwide even though a high proportion of individuals in developed countries have been vaccinated with vaccines that protect from severe symptoms. COVID-19 vaccines currently in use are based on the S antigen delivered in adenoviral vectors, or as mRNA or recombinant protein formulations. Most are effective at containing the severity of the disease (22). mRNA vaccines are usually included in lipid nanoparticles and induce high protection levels (23–27). These vaccines require the strict maintenance of the cold chain (-80 or -20 °C), which is a drawback for delivery in developing countries. Adverse effects such as anaphylaxis and myocarditis of these mRNA vaccine formulations are rare (reviewed in (22)). Non-replicating viral vector vaccines also require the cold chain (2-8 °C) and cause rare adverse events, such as thrombosis with thrombocytopenia and the Guillain-Barré syndrome, and proinflamatory response (22, 28). Second generation vaccines based on recombinant proteins with adjuvants lead to high specific antibody responses but confer low T-cell activation levels (29, 30). Although existing vaccines protect against severity of clinical signs, currently dominant variants, such as Omicron (B.1.1.529), are not very sensitive to these vaccines (5). Additional vaccine doses will unlikely tackle the problem of vaccine escape variants (31). New vaccines providing protection against a broader spectrum of VOCs are required. DNA vaccines are a promising method for COVID-19 control given their ability to be efficiently modified to achieve protection against new variants, their low cost, the relatively simple manufacturing process, and their thermotolerance, which would facilitate worldwide distribution (32). Low-income countries would particularly benefit from this vaccine because the cold chain isn’t required for storage and distribution (12). In this study, we describe a DNA vaccine against SARS-CoV-2 delivered by *in vivo* electroporation after injection *via* the IM route. This vaccine elicits a robust SARS-CoV-2-specific humoral and cellular immune response that protects mice from a lethal challenge with the Wuhan strain and the Delta variant.

The N protein in coronaviruses is a critical structural protein (33, 34) whose genetic stability and conservation among coronaviruses, including new putative variants, make it a suitable vaccine candidate (34). The S gene was modified to Sfs to avoid furin cleavage. HEK293 cells transfected with pPAL-Sfs + pPAL-N properly express the codon-optimized Sfs and N genes from the Wuhan strain. C57BL/6J mice vaccinated with two doses of pPAL-Sfs + pPAL-N by electroporation induced a robust humoral response, producing specific IgGs against the S protein and its RBD domain. Anti-RBD IgG subclass analysis showed the predominance of IgG2c, indicating a Th1/Th2 balance skewed towards Th1. Most of the asymptomatic SARS-CoV-2 infected mice exhibited an effective and robust Th1 response, which favors infection clearance (35). Several studies have shown that the initial establishment of a robust Th1 immune response leads to control of viral replication, whereas a potent Th2 response correlates with severe forms of the disease (36, 37). In addition to the generation of anti-S IgG antibodies, we have demonstrated that immunization with pPAL-Sfs + pPAL-N also induced production of neutralizing antibodies. Virus neutralization is a measure of antibody efficacy (38) and a correlate of vaccine-induced protection (39). Therefore, pPAL-Sfs + pPAL-N induces a humoral response compatible with protection against SARS-CoV-2.

T-cell responses likely play a significant role in SARS-CoV-2 infection. Strong T-cell responses correlated with recovery of patients that suffered mild disease (40, 41). A robust CD8^+^ T-cell response with broad specificity is considered a signature of successful protective immunity against SARS-CoV-2 (42). Patients who tested positive for CD8^+^ T cells are less sensitive to reinfection (19), highlighting the need for vaccines that elicit a potent cellular immune response. One of the main advantages of DNA vaccines is their ability to stimulate a strong T-cell immune response. Vaccination with pPAL-Sfs + pPAL-N activates specific T-cell responses to the SARS-CoV-2 S and N proteins. We assessed four functional parameters: CD107a, IFN-γ, IL-2, and TNF-α, to better understand the function and phenotype of the anti-S and anti-N T cells elicited by vaccination. The finding that cytotoxic CD4^+^ T cells are present in vaccinated mice is consistent with past studies (20). Cytokine profile analysis of antigen-specific CD4^+^ and CD8^+^ T cells indicated that pPAL-Sfs + pPAL-N vaccination induced polyfunctional T cells to different extents depending on the T cell compartment and the antigen. Very few activated cells expressing the four parameters were detected. Nonetheless, we found that more than 50% of CD8^+^ T cells specific to the S peptide pool were positive for two or more functional parameters, which indicates that these cells are polyfunctional effectors. Curiously, anti-N CD8^+^ T cells were less polyfunctional and most exclusively displayed a cytotoxic phenotype. Similarly, both anti-S and anti-N CD4^+^ T cells showed limited polyfunctionality. Still, both cell populations expressed CD107a in response to peptide stimulation, which demonstrates that these CD4^+^ T cells possess a cytotoxic phenotype. The presence of cytotoxic CD4^+^ T cells specific for SARS-CoV-2 in murine models has been reported (20), and it correlates with protection in some viral infections, such as West Nile fever (43). In future work, it would be interesting to determine to which extent these cytotoxic anti-SARS-CoV-2 CD4^+^ T cells contribute to protection. Overall, the data indicate that vaccination induces cellular immune responses against the SARS-CoV-2 antigens included in the vaccine formulation and that anti-S CD8^+^ T cell effectors are polyfunctional. Since T cell polyfunctionality can be a correlate of protection (44), pPAL-Sfs + pPAL-N vaccination could activate potent T cell effectors capable of recognizing the viral infection.

As previously stated, the pPAL-Sfs + pPAL-N vaccine triggers specific T-cell activation against N. In some viral infections, non-neutralizing antibodies directed to the nucleoprotein can help clear the infection of enveloped viruses (45–47). One of the mechanisms behind the protective role of these anti-N antibodies has been recently described (15). The E3 ubiquitin ligase TRIM21 may target the N protein for proteasomal degradation through anti-N antibodies, thus triggering the activation of effective cytotoxic T-cell responses against the N antigen (15). This suggests that including the SARS-CoV-2 N gene in the vaccine formulation may improve protection as it could provide T cell reactivity against this highly conserved antigen through multiple mechanisms.

The pPAL-Sfs + pPAL-N vaccine was capable of protecting mice against the Wuhan strain and the Delta VOC. Therefore, the humoral and cellular immune response triggered against the S and N antigens of SARS-CoV-2 by pPAL-Sfs + pPAL-N was likely capable of protecting mice from the disease. Our viral load analysis by qRT-PCR and PFU titration in lung, heart, and brain tissue homogenates indicate that pPAL-Sfs + pPAL-N vaccination prevented the virus from spreading to organs, such as the heart (19) or the brain, linked to serious long-term side effects of COVID-19. Noteworthy, protection was achieved in all vaccinated animals, including those with low specific antibody titers, indicating that the pPAL-Sfs + pPAL-N vaccine produces humoral and cellular immune responses, thus enhancing protection. Additionally, protection was achieved in all vaccinated animals when the SARS-CoV-2 challenge was performed 3 months after the last vaccination. Hence, pPAL-Sfs + pPAL-N vaccination induces long-term immunity against the disease and fully protects mice from the SARS-CoV-2 Wuhan strain and the Delta VOC. Therefore, the pPAL-Sfs + pPAL-N vaccine is ready to advance to human clinical trials.

In summary, homologous prime/boost administration of sterile-water-dissolved pPAL-Sfs + pPAL-N (20 µg per plasmid per dose) administered by the IM route and applying subsequent *in vivo* electroporation fully protects mice against challenge with 10^5^ PFU of the Wuhan-Hu-1 MAD6 isolate and the Delta variant. pPAL-Sfs + pPAL-N triggers a Th1 cell response, a polyfunctional CD8^+^ cytotoxic T-cell response, and the production of neutralizing antibodies. This DNA vaccine is safe, easy to produce at the industrial scale, and suitable for distribution and storage at room temperature.

## Data availability statement

The original contributions presented in the study are included in the article/**Supplementary Material**. Further inquiries can be directed to the corresponding authors.

## Ethics statement

The animal study was reviewed and approved by Consejo Superior de Investigaciones Científicas (CSIC) Ethics Committee and the Comunidad Autónoma de Madrid (Proex 254.6/21 and 295.6/21).

## Author contributions

PA, NS, and VL contributed to conception and design of the study. PA, JL, DR-M, AA, FL, JR, SR-G, AL-L, AC, PS-C, VM and NS performed the experiments. NS, VL, PA, AA and JR wrote the first draft. FL edited the manuscript. All authors contributed to the article and approved the submitted version.

## Funding

This work was funded by PTI-Salud Global (CSIC), Center for Technological and Industrial Development (CDTI), REACT-ANTICIPA-UCM (Comunidad de Madrid), and European Research Council (Advanced Grant VERDI, ERC2015AdG grant number 694160).

## Acknowledgments

We would like to thank Mercedes Domínguez and Inmaculada Moreno (Centro Nacional de Microbiología, Virología e Inmunología Sanitarias, Instituto de Salud Carlos III) for providing the polyclonal anti-N antibody, Luis Enjuanes and Juan Francisco García-Arriaza for providing the SARS-CoV-2 Wuhan-Hu-1 MAD6 isolate and the SARS-CoV-2 B.1.617.2 Delta variant, and Luis Enjuanes for providing Calu3 cells. We also acknowledge Angélica Horrillo, María Herrero, and Marta Cerceda (CIBMS-CSIC) for assistance in animal experimentation.

## Conflict of interest

The authors declare that the research was conducted in the absence of any commercial or financial relationships that could be construed as a potential conflict of interest.

## Publisher’s note

All claims expressed in this article are solely those of the authors and do not necessarily represent those of their affiliated organizations, or those of the publisher, the editors and the reviewers. Any product that may be evaluated in this article, or claim that may be made by its manufacturer, is not guaranteed or endorsed by the publisher.
