# Supplementary material for: Non-replicative antibiotic resistance-free DNA vaccine encoding S and N proteins induces full protection in mice against SARS-CoV-2
Source: Front Immunol. 2022 Nov 9;13:1023255. doi: 10.3389/fimmu.2022.1023255 (PMC9682132; doi:10.3389/fimmu.2022.1023255)
Supplement: Supplementary file 1 [file Table_1.pdf]

Table S1. Clinical score evaluation in SARS-CoV-2-infected K18-hACE2 mice.

Observation	Score
**Body weight**<br>- No change<br>- Loss of body weight in % = score points (e.g. loss of 8% body weight = 8 points)<br>- Loss of >25%	 0<br>1-25<br><br>25
**Appearance**<br>*Fur*<br>- Shinning<br>- Matte<br>- Ruffled<br>*Eyes*<br>- Clear<br>- Unclean, sticky, closed, semi-closed<br>*Posture*<br>- Normal<br>- Hunched<br>- Massively hunched	 <br>0<br>2<br>5<br><br>0<br>5<br><br>0<br>5<br>15
**Motility**<br>- Spontaneous (normal behaviour + social contact)<br>- Spontaneous but reduced<br>- Moderately reduced<br>- Motility only after stimulation<br>- Coordination disorder<br>- Lethargy	 0<br>1<br>5<br>15<br>20<br>25
**Respiration**<br>- Breathing normal<br>- Breathing slightly changed<br>- Accelerated breathing (>30%)<br>- Strongly accelerated breathing (>50%)	 0<br>1<br>10<br>25
**Severity rating**	**Sum score**
Severity level 0: No burden, animals are healthy<br><br>*Severity level 1:* Low disease burden<br><br>*Severity level 2:* Moderate disease burden<br><br>*Severity level 3:* Moderate to severe sickness<br><br>*Severity level 4:* Severe burden (Implement human endpoint)	0-5<br><br>5-10<br><br>10-25<br><br>25-50<br><br>>50
